# Mummification in Korea and China: Mawangdui, Song, Ming and Joseon Dynasty Mummies

**DOI:** 10.1155/2018/6215025

**Published:** 2018-09-13

**Authors:** Dong Hoon Shin, Raffaella Bianucci, Hisashi Fujita, Jong Ha Hong

**Affiliations:** ^1^Lab of Bioanthropology, Paleopathology and History of Diseases, Department of Anatomy/Institute of Forensic Science, Seoul National University College of Medicine, Seoul 03080, Republic of Korea; ^2^Legal Medicine Section, Department of Public Health and Pediatric Sciences, University of Turin, Italy; ^3^Warwick Medical School, Microbiology and Infection Unit, The University of Warwick, Coventry, CV4 7AL, UK; ^4^UMR 7268, Laboratoire d'Anthropologie Bio-Culturelle, Droit, Etique et Santé (Adés), Faculté de Médecine de Marseille, France; ^5^Department of Bioanthropology, Niigata College of Nursing, Joetsu, 943-0147 Niigata, Japan

## Abstract

Over the decades, mummy studies have expanded to reconstruct a multifaceted knowledge about the ancient populations' living conditions, pathologies, and possible cause of death in different spatiotemporal contexts. Mainly due to linguistic barriers, however, the international knowledge of East Asian mummies has remained sketchy until recently. We thus analyse and summarize the outcomes of the studies so far performed in Korea and China in order to provide mummy experts with little-known data on East Asian mummies. In this report, similarities and differences in the mummification processes and funerary rituals in Korea and China are highlighted. Although the historical periods, the region of excavation, and the structures of the graves differ, the cultural aspects, the mechanisms of mummification, and biological evidence appear to be essentially similar to each other. Independently from the way they are called locally, the Korean and Chinese mummies belong to the same group with a shared cultural background.

## 1. Introduction

 The dead do speak and mummies speak up. Through a comprehensive and holistic approach to the civilizations of the past, scholars aim at tracing the biological and sociocultural profiles of ancient populations back. Over the decades, the living conditions, pathologies, and possible cause of death of ancient populations in different spatiotemporal contexts (i.e., ancient Egyptians mummies, bog bodies, the Similaun Man (Oetzi), crypt mummies, the Arctic and high-altitude permafrost mummies, and South American precontact mummies) were progressively reconstructed by mummy studies [1–3].

East Asian countries have created rich cultural heritages in the continent for a long time. Over the past 60 years, several important studies were also carried out on East Asian mummies and meaningful achievements were reached. Although East Asia is not a region where a large number of mummies are found, researchers have extensively studied these remains and have released valuable academic reports. Due to language barriers, however, most reports were not widely and efficiently diffused to English-speaking academia. From an academic point of view, scholars outside of Asia were unable to comprehensively understand the complexity of these studies.

Actually, the reality of the academic tradition of mummy studies in East Asia is distinct from other continents. East Asian mummies are culturally and biomedically so unique that extensive dissemination of cutting-edge research is paramount. Except for short introductions and sporadic reports [2, 4, 5], however, a review containing perfect data about the East Asian mummies is still lacking. And western researchers' knowledge of East Asian mummies has remained sketchy until recently. Here we thus analyse and summarize all previous studies written in Korean, Chinese, and English so to provide mummy experts with little-known information to date.

## 2. The Spatiotemporal Scope of This Review

**Figure 1:**
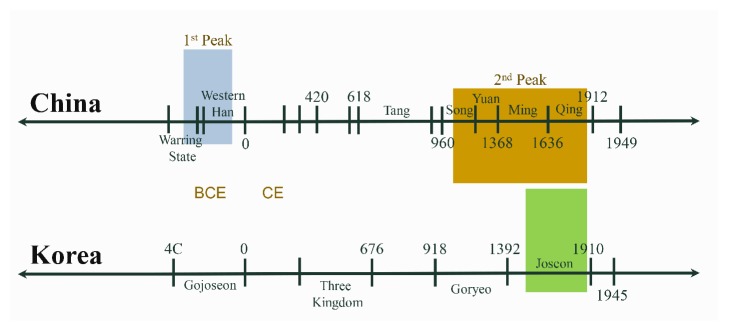
Mummies of China and Korea in the historical frame. There are two peaks in the discovery of Chinese mummies. The first peak (shaded in blue) corresponds to the Warring States (402-221 BCE) and Western Han Periods (202-8 BCE); the second peak (shaded in brown) dates to the Song (960-1279 CE) and Ming Dynasties (1368-1644 CE). The box shaded in green indicates the South Korean mummies of the Joseon Dynasty (1392-1910 CE).

East Asia includes the countries, districts, or municipality of Korea, Japan, China, Mongolia, Taiwan, Macau, and Hong Kong. However, this review will focus mainly on the mummies discovered in Korea and China. Here we examined the Chinese mummies of the Warring States (402-221 BCE) and Western Han Periods (202-8 BCE), those discovered in the tombs of the Song (960-1279 CE) and Ming Dynasties (1368-1644 CE) and the 16^th^ to 18^th^ South Korean mummies of the Joseon Dynasty (1392-1910 CE) (Figure 1). As to the ancient and excellently preserved mummies from the Tarim Basin (northwest China), their description will not be included in the present review. Given the complexity of their cultural background and antiquity (1800 BC to the first centuries BC), a separate analysis is required.

## 3. The 16^th^ to 18^th^ Century Korean Mummies of Joseon Dynasty

Over the last decade, archaeological excavations carried out in South Korea have led to uncover several 16^th^ to 18^th^ century mummies buried in their original graves (Figure 1). Interdisciplinary studies [6–8] were performed on mummified tissues and organs; and precious information was gained on the lifestyle and pathologies of premodern Korean people.

**Figure 2:**
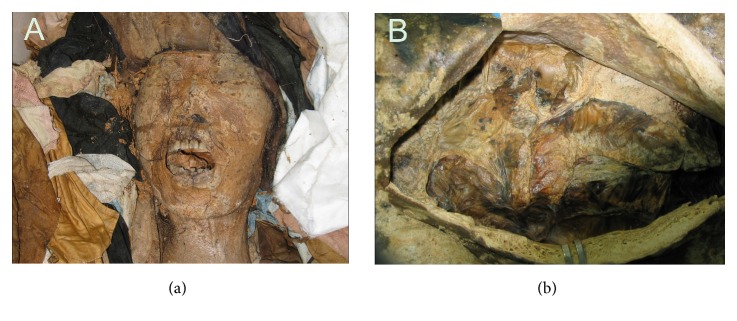
Perfectly preserved Korean mummies (Gangneung). (a) Skin and hair were intact. (b) Mummified intestines were perfectly preserved.

Anatomical, histological, and radiological techniques were applied to verify the state of preservation of the Korean mummies (Table 1; Figure 2). According to anatomical examination, skin and hair were perfectly preserved. Histology revealed that Korean mummies' hard and soft tissues showed an excellent state of preservation. The most common histological component observed in the mummified organs was represented by collagen fibers; nevertheless, cell debris of red blood cells, chondrocytes, hepatocytes, and muscle cells were also identified. Although the brain size had shrunken, the organ still kept its original morphology. Brain tissue histology showed that myelin remnants were mainly preserved components [9–12]. Lim et al. [13] found that autofluorescence was emitted from cell residues preserved in some tissues, a finding which was interpreted with great care. As a matter of fact, during microscopic observation, it is important to ascertain whether the immunofluorescence observed in ancient tissues is specific or not [13].

In Korean mummy researches, radiology showed to be a highly efficient diagnostic tool [14] that enabled researchers to establish the state of preservation of the inner organs and to estimate the patient's pathological conditions in a noninvasive way. However, the radiological approach also has its own biases. Since mummified tissues and organs underwent taphonomic changes over the centuries, it may be difficult to apply modern radiological knowledge to ancient bodies. To overcome these biases (pathology versus pseudopathology),* post factum* dissections were performed to confirm the actual pattern of the mummified organs previously observed by computed tomography (CT) [15]. Magnetic Resonance Imaging (MRI) was also applied on a hydrated Korean mummy, providing researchers with invaluable information on the state of preservation of the organs with minimal damages [16]. Lastly, endoscopy showed that the organs of the Korean mummies displayed a “vivid” appearance though Kim et al. [17] were skeptical about the real efficiency of this minimally invasive technique applied to the study of ancient bodies.

Mummies have been a valuable source of information on the diseases that plagued the ancient Korean people (Table 2). For instance, atherosclerotic cardiovascular disease was confirmed in a 17^th^ century Korean mummy by anatomical [18] and paleogenetic techniques [19]. Kim et al. [20] identified calcified pulmonary nodules in a 350-year-old-Joseon mummified individual, thus providing scholars with the oldest evidence of ancient pulmonary tuberculosis in South Korea. Thanks to multiple biomedical techniques, congenital diaphragmatic hernia [21] and Cherubism [22] were also diagnosed in Korean mummies.

Research on ancient parasites was a particularly successful field of investigation. Since the first paleoparasitological report performed on a child mummy [23], remarkable evidence of ancient parasitism was accumulated through multiple studies. Using light and electron microscopy, Shin et al. [24] showed an excellent state of preservation of ancient parasite eggs in coprolites. To date (December 2016), the paleoparasitological studies were conducted on coprolites from 24 Korean mummies, allowing the parasite infection prevalence of 16^th^ to 18^th^ century Joseon people [25, 26] to be estimated. The prevalence of soil-transmitted parasites among the 16^th^ to 18^th^ century Joseon mummies was estimated to be 58.3 % for* Ascaris *sp. and 83.3 % for* Trichuris *sp.; and this prevalence is quite similar to the one described in the 1971 Korean National Survey. The infection rate of soil-transmitted parasites dropped with the rapid industrialization occurred during the 1980s [26]. More specifically, concerning the Trematode species, the Joseon mummies showed very high infection rates (25 % for* Clonorchis*; 33.3 % for* Paragonimus*) whereas only 4.6 % (*Clonorchis*) and 0.09 % (*Paragonimus*) infection rates were detected in the 1971 National Survey [26]. This implies that the Trematode infection rates had already decreased way before the beginning of modernization in South Korea whereas the changing pattern of the infection rates of soil-transmitted parasites in South Korea occurred around the time of modernization [26]. Why the Trematode infection rates varied before the modernization has still to be determined.

Cases of parasitism rarely seen among clinical patients were reported in the Korean mummies. For examples, ectopic (hepatic) paragonimiasis was identified in a 17^th^ century Korean mummy [27]. A liver mass just underneath the diaphragm was identified through CT scanning; a subsequent microscopic examination revealed the presence of multiple ancient* Paragonimus* sp. eggs inside the mass. Actually, this was the first archaeoparasitological case of liver abscess caused by ectopic paragonimiasis. Another case of ectopic paragonimiasis was also observed in 400-year-old Korean female mummy [28]. Here,* Paragonimus* eggs were detected in lung, feces, intestine, and liver samples, but not in the brain nor in pelvic-cavity-debris (Table 2). The repeated reports of ectopic paragonimiasis indicate that the disease was widespread in the Korean people during the Joseon period.

*Gymnophalloides seoi* infection was also a unique paleoparasitological finding.* G. seoi* eggs were detected in specimens from two different mummies discovered on the coastal areas of the Korean peninsula [25]. Considering that* G. seoi* is not currently endemic in the counties and that the endemic focus is confined to a restricted portion in the coastal areas of the Korean peninsula [25], we speculate that this parasitic infection was more widespread during the Joseon period.

Ancient DNA (aDNA) analysis has become an important tool for revealing the phylogenetics of pathogens and the genetic profiles of the deceased. Ancient DNAs of Hepatitis B virus,* Helicobacter pylori, Ascaris *sp*., Paragonimus westermani, Clonorchis sinensis *(*C. sinensis*), and* Trichuris trichiura *(*T. trichiura*) were sequenced [28–35]. Single Nucleotide Polymorphisms were analyzed to identify ABCC11, EDAR, FGFR2, and ABO genotypes [36]. In addition, various studies such as palynological analysis [37] or forensic craniofacial reconstructions [38] were successfully performed.

## 4. Why Did the Korean Mummies Spontaneously Preserve?

Which kind of mummification allowed the Korean mummies to preserve? Climate in Korea is not suitable for natural mummification and, before the 20^th^ century, the Joseon did not resort to embalming techniques [42]. Cultural beliefs implied that the intact preservation of the ancestors' corpses was an ominous sign for the descendants. In this regard, the discovery of a series of perfectly preserved mummified bodies became a sensational topic in South Korea.

**Figure 3:**
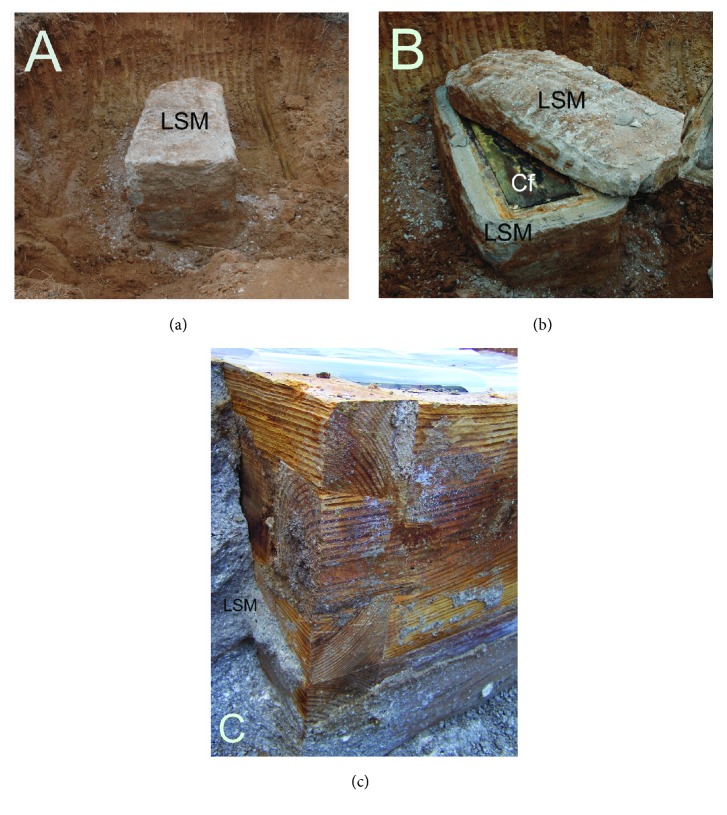
(a) and (b) An example of complete* lime soil mixture* (LSM) sealing around the coffin (Cf) of Joseon grave discovered at the Hadong archaeological site, South Korea. (c) Perfectly preserved coffin wood.

**Figure 4:**
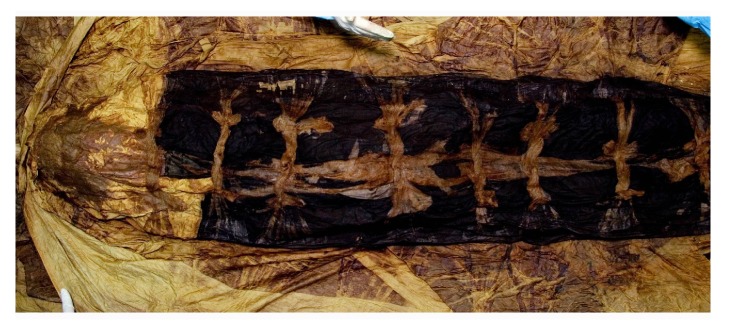
Clothing wrapped around the Korean mummy was found inside the Joseon grave (SN1-2).

Actually, the mummification process was not likely to be induced solely by natural or artificial causes, but is more likely the result of multiple, complex and synergic mechanisms. Korean researchers interested in the actual mechanism of mummification paid attention to the unique structure of the graves (called* Hoegwakmyo *or* the grave with lime soil mixture barrier*) where the Joseon people had been laid to rest [43]. During the Joseon period, lime, red clay, and sand (called* sammul *or* lime soil mixture*) were blended together in given proportions to construct the* Hoegwakmyo* tomb. The mixture was poured around the coffin and, once hardened, it completely sealed the grave (Figure 3). Since the Korean mummies were rarely found in partially or totally destroyed* Hoegwakmyo* graves, it can be inferred that the sealing itself played a major role in promoting the mummification [44]. It was also noted that a large amount of clothing was used to fill the coffins [7] (Figure 4). The use of textiles combined with the sealing produced a shortage of oxygen inside the coffin.

Animal experiments were carried out to reproduce the mummification process. Rats and miniature* Hoegwakmyo* grave models were used. It was observed that while there is hardening around the coffin, the* lime soil mixture* generated high temperatures. Since the heat lasted for quite a long time, it completely killed the bacteria in the animal intestines, promoting a successful mummification. Although the experiment was performed on an animal model using a miniature coffin, it is likely that a similar phenomenon occurred in the actual-sized* Hoegwakmyo* graves [42, 44]. Taken together, the use of a* lime soil mixture*, the textile filling, the low content of oxygen inside the coffin, and the high temperature produced by the hardening of* lime soil mixture* operated in synergy and enhanced the mummification process.

According to historians, the emergence of the* Hoegwakmyo* grave is closely related to the reform of the funeral rituals pursued by the Confucianists of the Joseon Dynasty (1392-1910 CE). The Confucianist ruling class people, who had overthrown the Goryeo Dynasty (918-1392 CE), believed that the funeral rituals had been seriously polluted by Buddhists' ideals. Therefore, the funerary rituals were modified and adapted to the teachings of the Confucianism masters. The Confucianist ritual book,* Jujagare *(*Zhouzijiali *in Chinese), was written by the great Chinese scholar master,* Zhu Xi* (1130-1200 CE). The introduction of the book in Korea deeply influenced the funeral rites of the Joseon kingdom. In the* Jujagare*, the* Hoegwakmyo *tomb was recommended as the ideal Confucianist gentlemen's burial. The ruling class of the Joseon Dynasty assimilated this concept and the* Hoegwakmyo *tomb became their elective type of grave [5, 43]. However, what has become also evident nowadays is that the Joseon people did not want their corpses to be mummified. In that sense, the mummification in the* Hoegwakmyo* graves was a kind of unexpected accident.

## 5. Mummies of* Sticky Rice Soup Sealed Tombs* in China

**Figure 5:**
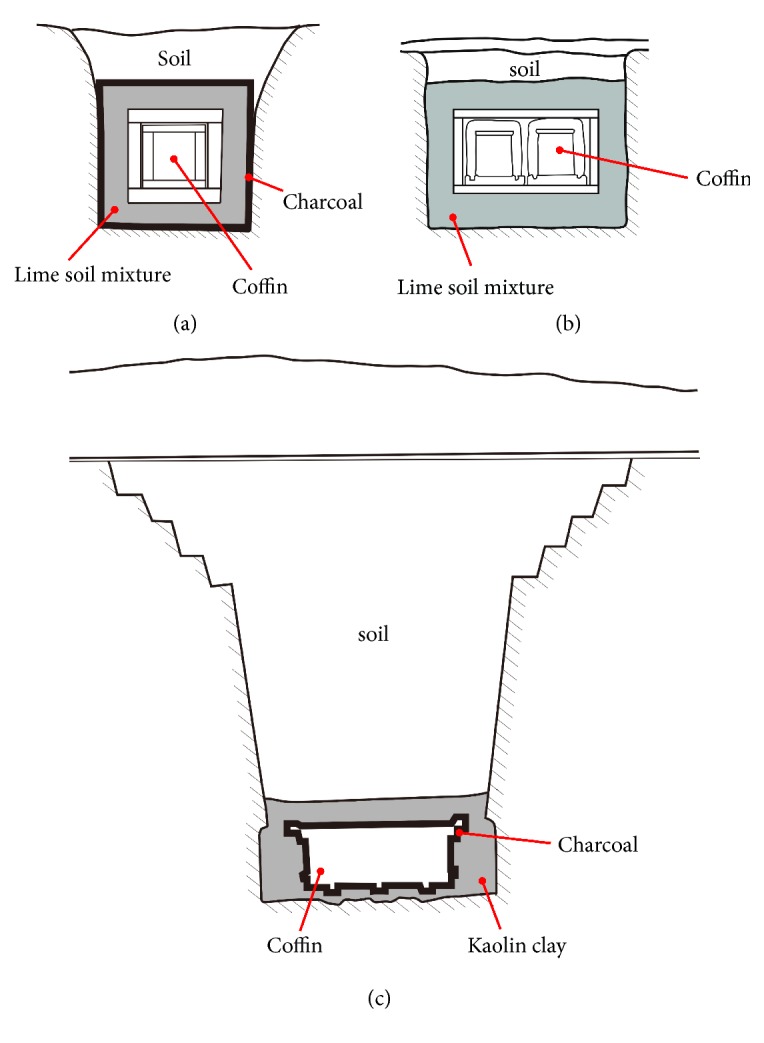
Tomb structures: (a) Korean* Hoegwakmyo* of Joseon Dynasty [39]; (b) the* sticky rice paste (or soup) sealed tomb* of Song-and-Ming Dynasty, China [40]; (c)* Mawangdui* grave of Western Han Dynasty [41]. Kaolin clay in (c) possibly played the role of sealant performed by the* lime soil mixtures* in (a) and (b). The figures herewith were redrawn based on the previous reports about* Hoegwakmyo* [39], Song-and-Ming Dynasty tomb [40], and* Mawangdui* grave [41].

Since there is a close relationship between the ancient Korean people and Confucianism, researchers hypothesized that similar tombs and mummies were present in China, the country where Confucianism originated [5]. Interestingly, Chinese archaeologists reported that some graves dating to the Song and Ming dynasties were completely sealed by a mixture of lime, yellow clay soil, sand, and sticky rice water. These tombs were called ‘*sticky rice paste (or soup) sealed tomb'* [5, 45]. In this regard, the Korean* Hoegwakmyo* tomb was very similar to the Chinese ‘*sticky rice paste (or soup) sealed tomb' *(Figures 5(a) and 5(b)).

May the* sticky rice paste (or soup) sealed tomb* be considered the prototype of the Korean* Hoegwakmyo*? According to the Chinese archaeological reports, the earliest* sticky rice paste (or soup) sealed tomb *so far discovered is the* Sun Siniangzi mu* (tomb) in the city of Jiangyin (Jiangsu province). The grave contained the mummified body of the wife of a Northern Song Dynasty (960–1127 CE) bureaucrat [5, 46]. Since the* Sun Siniangzi mu* was constructed in 1055 CE, the origin of the* sticky rice paste (or soup) sealed tomb *can be placed at the beginning-middle 11^th^ century.

If this type of graves was related to the* Hoegwakmyo* tombs of the Joseon Dynasty, these findings would have implications also in tracing the origin of the Korean mummies back. It has to be underlined that the Chinese* sticky rice paste sealed tombs* were not identical to each other and varied in shape [5]. From this perspective, only a part of them showed similarities with the 16^th^ to 18^th^ century Joseon* Hoegwakmyo* graves. This implies that when the use of the* Hoegwakmyo *graves emerged for the first time in the Korean history, only a certain type of the Chinese* sticky rice paste sealed tombs* was selectively introduced in the Joseon society. Therefore, the understanding of the history of the Chinese tombs provides scholars with additional information on the history of the Korean* Hoegwakmyo *tombs.

Various biomedical studies were performed on the mummies exhumed from the* sticky rice soup sealed tombs*. In general, these corpses were very well preserved, wet-type mummies. Researchers observed that the Korean and Chinese mummies shared several common features. Both had elastic skin and flexible joints; hair, nails, and teeth showed a good state of preservation. The inner organs were fairly well preserved. These features were particularly evident in the case of the* Xu Fan* couple found in a tomb of the Ming Dynasty [5, 40]. Histology showed intact, well-defined cartilage cells and nuclei in the mummified tissues of both individuals. Paleopathological investigations showed that the husband (*Xu Fan*) suffered from atherosclerosis and coronary artery disease [5, 40].

Another interesting case was that of the 13^th^ century* sticky rice paste sealed tomb *containing the mummified body of the wife of the official* Wu Chou*. The mummy belonged to the Southern Song Dynasty and was found in 1988 in the De'an County (Jiangxi province). The grave dated to 1274 CE [5, 47]. A circa 30 centimetres thick* lime soil mixture* layer was present around the coffin. At the time of discovery, the body, originally wrapped in silk, was found flooding in the water rising from the floor of the coffin. The preservation of the mummy was excellent. The lady was 152 cm tall [5, 47]. In terms of quantity and quality, the scientific information gained from the studies performed on the Chinese mummies was excellent and comparable to the achievements obtained by the study of the Joseon mummies.

The most advanced part of the scientific study on the Song-and-Ming dynasty Chinese mummies is the paleoparasitological one. As early as 1956,* Ascaris *eggs were identified in a coprolite from a 450-year-old male mummy belonging to the Ming dynasty. Ancient parasite eggs of* Ascaris lumbricoides*,* Fasciolopsis buski*,* C. sinensis*, and* T. trichiura *[48, 49] were also found in mummies of the Song-and-Ming Dynasties. The overall pattern of the paleoparasitological studies looks very similar to those carried on the Korean mummies.

What kind of mummification occurred inside* the sticky rice soup sealed tomb*s? Chinese archaeologists proposed that multiple factors such as the complete sealing of the coffin by* lime soil mixture* (Figure 5(b)), the constant temperature/humidity inside the coffin, and other minor factors were responsible for the excellent mummification [5, 40, 50]. Again, densely packed clothing was found inside the Chinese coffins. When clothes are filled up tightly leaving no empty space inside the coffin, bacteria are unable to proliferate and die; thus, mummification occurs [5, 45, 50]. Moisture absorbent, such as charcoal, put inside the coffins and the bactericidal effect of lime may have been also involved in the mummification processes occurring in the* sticky rice paste sealed tombs *[5, 45, 50].

In brief, the Chinese* sticky rice paste sealed tombs* and the* Hoegwakmyo *of the Joseon society share many features, i.e., the structure of the coffin, the presence of* lime soil mixture* layer placed around the coffin, and the use of heavily packed clothing.

## 6. Chinese Mummies of Warring States and Western Han Period

While Korean scholars paid attention to the possible links between the mummies exhumed from the* sticky rice soup sealed tombs *and those from the Joseon* Hoegwakmyo*, Chinese scholars focused on the similarities existing among the Chinese mummies. More specifically, similarities were identified between the mummies from the* sticky rice soup sealed tombs* and those exhumed from the Warring States (402 BCE - 221 BCE) and Western Han Period (202 BCE – 8 CE) graves.

Among the Warring States and Western Han period graves so far investigated, only three corpses were classified as mummies. These corpses, which have been thoroughly studied, are currently displayed in the Hunan and Hubei provinces of China. Detailed information concerning these mummies is summarized in Table 3.

Actually, the graves of the Warring States Period (402 BCE-221 BCE) were discovered in the territory of Chu, an ancient kingdom that prospered in the present Yangtze River basin. In February 1994, grave robbers plundered a Chu tomb (*Guo-Jia Gang Tomb No. 1*) located in the city of Jingmen (Hubei province) [4, 51]. Many cultural artefacts were damaged or lost. Quite luckily, a female mummy, almost undamaged, was recovered. According to the archaeologists, the grave was constructed before Qin's unification of China (221 BCE), more precisely in the middle stage of the Warring States Period. This implies that this individual, whose corpse was buried in the tomb at least 2,300 years ago, represents the earliest case of mummification ever reported in East Asia to date [52].

According to the archaeoanthropological reports, a duplicated coffin (an outer and an inner coffin) was used for her burial and the coffin was found at circa 7 meters below the soil level. The state of preservation of the mummy was perfect. Aged 70-75 at death, the woman was 160 cm tall. Her blood type was AB. Parasitology revealed that she was infected by* C. sinensis* and* T. trichiura *[52]. The mummy is currently displayed in* Jingmen Museum*.

Apart from the Chu female mummy, another tomb called* Mawangdui (Mawangtui) *grave provided scholars with an exceptional finding. In 1971, during the construction of an air-raid shelter, a grave of the Western Han period was discovered at a depth of circa 20 meters. The archaeologists, who successfully excavated the tomb in a period of political constraints, found multiple coffins (two outer and four inner coffins) of different sizes fitted one within another. When the innermost coffin was opened, the archaeologist discovered the ‘*cadaver*' of a woman that did not show evidence of decomposition [4]. According to archaeologists, at the time of discovery, the mummy was flooding in a liquid that filled the coffin.

The lady's name was confirmed to be* Xin Zhui*, the wife of* Li Cang* (or* Li Tsang*), Marquis of Dai (or Tai) during the Western Han Period. Since she died in 168 BCE, she must have been buried about 100 years later than the above-mentioned* Jingmen* mummy [4]. After two thousand years, the mummified lady and her tomb assemblage were amazingly well preserved. Researches performed on the tomb assemblage found in the* Mawangdui* grave provided scholars with valuable information about the life of this ancient Chinese lady [2].

The* Mawangdui* mummy underwent thorough biomedical investigations [53, 54]. The body of the lady, who was 154 cm tall, weighted 34.3 kg. Her blood type was A. Her skin and hair were intact, soft tissues had maintained the original elasticity, and the joints could be moved freely. X-rays showed that the skeleton was complete. At autopsy, it was shown that although the inner organs were remarkably shrunken, their relative positions had remained unaltered. Histology showed that both peripheral nerves and skeletal muscles were well preserved [55]. Many signs of ancient diseases were identified in the* Mawangdui* lady: atherosclerosis, coronary artery disease, cholelithiasis (gallstones), lead and mercury chronic poisoning, and fracture and malunion of the distal end of the right ulna and radius. Based on the pathological evidence, it was hypothesized that the most likely cause of death was a myocardial infarction or an arrhythmia due to heart attack possibly consequent to a biliary colic [4, 56]. Muskmelon seeds (n=138.5) were found inside her intestines and paleoparasitology showed that she had suffered from* Schistosoma japonicum*,* T. trichiura*, and* Enterobius vermicularis* [49]. All these studies provided scholars with unexpected information about the life of a 2,000-year-old Chinese woman. The mummy is currently displayed in* Hunan Museum*, along with other artefacts.

Another mummy of the Western Han period was discovered in 1975. The mummy was uncovered in a Western Han Dynasty grave (*Phoenix Hill No. 168*) in the Jiangling County (Hubei Province) [57]. According to the archaeologists, a triplicated coffin (one outer and two inner coffins) was identified approximately 10 meters underneath the soil surface [4, 58]. Similar to the* Mawangdui's *Lady, the corpse was immersed in dark red fluids (100 litters at a depth of 75 cm) [4]. The body belonged to a male, named Sui, an official (*wutaifu or wutafu*) of Western Han Dynasty who had died in 167 BCE when he was ca 60 years old [4].

Anatomical and histological studies confirmed that the state of preservation of the mummy was excellent. Soft tissues had maintained their elasticity. No hair was preserved whereas all teeth were present. The body measured 167.8 cm and weighted 52.5 kg; the blood type was AB. Autopsy showed well-preserved inner organs. In general, histology showed that most cells had disappeared whereas the collagen fibers were still abundant. The main component of the nervous tissue was represented by myelin remnants [4, 59]. No osteoporotic changes were found. The man suffered of chronic cholecystitis, parasitic hepatic cirrhosis, gallstone, and atherosclerosis. The cause of death was attributed to acute peritonitis due to a chronic gastric ulcer at the lesser curvature of stomach complicated by acute perforation [2, 59, 60]. Parasitology showed the presence of ancient eggs of* S. japonicum*,* C. sinensis*,* Taenia* sp., and* T. trichiura* [2, 49, 59, 61]. Sui's mummified body is currently displayed in Jingzhou Museum.

The 2,000-year-old mummies of the Warring States (402 BCE - 221 BCE) and Western Han period (202 BCE – 8 CE) have a high reputation in China. The perfectly preserved artefacts recovered from their graves provided scholars with information that, otherwise, would not have been obtained through the historical and archaeological studies.

The three Warring States and Western Han mummies seem to share some factors that favoured their preservation. Besides temperature, humidity, pressure, and pH, more inducing factors were suggested to be responsible for such an excellent preservation. Among these factors are the air-tight sealing of the coffin, the depth of the burial (Figure 5(c)), and the presence of cinnabar (HgS) in the liquid found inside the coffins at the time of discovery [4, 59]. It is also noteworthy that charcoal and kaolin clay (Bai gao ni) were used in sealing the coffins [56]. Kaolin clay may have played the same role of sealant performed by the* lime soil mixture* in the* Hoegwakmyo *graves and in the* sticky rice paste sealed tombs *(Figure 5). The complete sealing of the Warring States-Western Han graves by kaolin clay may have prevented the percolation of water and air into the coffin, thus creating anoxic conditions inside the inner coffin and halting the decomposition [56, 62]. The charcoal layer of the* Mawangdui* tomb may have also contributed to the preservation of both mummy and artefacts by absorbing the moisture that otherwise would have seeped inside the coffin [56].

## 7. Mummies of Korea and China in East Asian History

As shown in the present review, the Chinese mummies date back mainly to two historical periods. The first period corresponds to the Warring States and Western Han Periods (circa 2,000 years ago). The majority of the mummies dating to these periods were uncovered in the Hubei and Hunan Provinces. For next almost 1,000 years, very few mummies dating to the first millennium CE were found in China. When the second millennium began and the Chinese constructed the* sticky rice paste sealed tombs *during the Song and Ming dynasties, mummified bodies were newly recovered.

As previously stated, the Korean mummies unearthed from the* Hoegwakmyo* graves appear to be closely related to the Chinese mummies found in the* sticky rice paste sealed tombs*. Interestingly enough, the Chinese scholars did not consider the mummies belonging to the two distinct historical phases (Warring States and Western Han period versus Song/Ming dynasties) as separate entities. Rather, the corpses were classified as* wet corpses* [50],* ancient corpses* [4], or just* cadavers* [4, 54, 56], thus emphasizing the differences between the Chinese mummies and the mummies from other continents. In China, the so-called* ‘Mawangdui type cadaver'* generally includes the Warring States and Western Han Period mummies as well as the Song/Ming period mummies [4]. In our opinion, the corpses exhumed from the two different Chinese historical phases and the Korean mummies should be included in the same category.

Actually, Korean and Chinese mummies share many cultural and biological characteristics. Researchers agreed that both the mummification and the artefacts' preservation in the* Mawangdui* like graves were most likely due to the presence of a kaolin clay layer surrounding the coffin [56, 63–65]. Wang and Zhang [50] also proposed that the complete sealing of the coffin obtained resorting to the* lime soil mixture* was an important inducing factor for mummification in the* sticky rice paste sealed tombs.* In brief, the complete isolation of the inner coffin, either with kaolin clay or* lime soil mixture*, was responsible for mummification both in China and in Korea. The funerary customs also played an important role in the mummification process. For example, the tight packing of the cloths inside the coffin was a shared funerary custom in China and Korea. And above all, both in Korea and in China, the tombs' construction followed the precepts of the Confucianist tradition.

From a biomedical perspective, the mummies of Korea and China show considerable similarities. In both countries, the dead body did not undergo embalming and the internal organs were not removed from the body cavities. The macroscopical and radiological appearance of the mummified organs was similar in the Chinese and Korean examined cases. All inner organs were considerably shrunken and distorted, displaced dorsally but fixed in their relative position. Also, the histological findings were similar in mummies from both countries. In the case of the* Mawangdui* type mummies, microscopy revealed that the collagen fibers were the best preserved component. A considerable number of chrondrocytes were also found in cartilages and myelin remnants were the predominant structures identified in the nervous tissue [4]. A closer look at the histology of the Korean mummies reveals a strict similarity with the Chinese* Mawangdui* type mummies.

## 8. Conclusions

Interdisciplinary researches performed on the Korean mummies have led to gain invaluable scientific information on the health and disease statuses of past populations. Thanks to a growing body of literature written in English, the international scientific community has rapidly recognized the distinctiveness of the Korean mummies. Conversely, except for some brief reports, the investigations performed on the Chinese mummies were seldom presented to the international academia [2, 5]. Nevertheless, since the 1970s, China had a long history of high quality research on mummified bodies. The present review contains a synthesis of the studies carried out so far on the Korean and Chinese mummies.

It is worth noting that strict cultural and biomedical similarities can be identified among the Chinese* Mawangdui* type mummies, the mummies of the Song-and-Ming dynasties (the* sticky rice soup sealed tombs), *and the Korean mummies of Joseon dynasties. Even if the historical periods, the regions of excavation, and the structures of the graves varied, similarities in the mummification processes and funerary rituals are also highlighted. In this study, this statement is also confirmed by biological evidence. Independently from the way they are locally called, the East Asian mummies belong to the same group with a common cultural background.

## Figures and Tables

**Table 1:** Joseon *Hoegwakmyo* graves investigated by interdisciplinary research.

**Mummy**	**Research institute concerned**	**Year **	**Discovered during**	**Sex**	**Conducted research**	**Preservation**
Kunkook	Dankook University	2001	Archaeological excavation	Female	Am, PP, PPr, CT, aDNA	Mummy

Yongin	Gyeonggi Cultural Foundation	2006	Archaeological excavation	Female	Am, PP, PPr, CT, aDNA	Half mummified

Hadong-1	Jinju National Museum	2006	Moving a grave	Female	Am, PP, PPr, CT, aDNA	Half mummified

Gangneung	Gangneung Choi clan	2007	Moving a grave	Male	Am, PP, PPr, CT, MRI, aDNA	Mummy

SN1-2	Hangang Institute of Cultural Heritage	2007	Archaeological excavation	Male	Am, PP, PPr, CT, aDNA	Half mummified

SN PK	Hangang Institute of Cultural Heritage	2007	Archaeological excavation	Male	Am, PP, PPr, CT, aDNA	Half mummified

GJ1-2	Chungnam Institute of History and culture	2007	Archaeological excavation	Male	Am, PP, PPr, CT, aDNA	Half mummified

Seocheon	Gyeonggi Cultural Foundation	2008	Archaeological excavation	Female	Am, PP, PPr, CT, aDNA	Half mummified

Waegwan	Daedong Institute of Cultural Heritage	2008	Archaeological excavation	Male	Am, PP, PPr, CT, aDNA	Half mummified

Dangjin	Chungnam Institute of History and culture	2008	Archaeological excavation	Female	Am, PP, PPr, CT, aDNA	Half mummified

Hadong-2	Descendant of Gangneung Onyang Jung clan	2009	Moving a grave	Female	Am, PP, PPr, CT, aDNA	Half mummified

Mungyeong	Gyeongju National Research Institute of Cultural Heritage	2010	Moving a grave	Female	Am, PP, PPr, CT, aDNA	Mummy

Jinju	Dong-Seo Institue of Cultural Heritage	2010	Archaeological excavation	Male	Am, PP, PPr, aDNA	Half mummified

Sapgyo	Chungcheong Institue of Cultural Heritage	2011	Archaeological excavation	Male	Am, PP, PPr, CT, aDNA	Half mummified

Sacheon	Gyeong-Sang Cultural Heritage Research Center	2011	Archaeological excavation	Female	Am, PP, PPr, aDNA	Half mummified

Hwasung	HanBeak Institue of Cultural Heritage	2012	Archaeological excavation	Male	Am, PP, PPr, aDNA	Mummy

YG2-4	Honam Institute of Cultural Heritage	2012	Archaeological excavation	Female	Am, PP, PPr, aDNA	Half mummified

YG2-6	Honam Institute of Cultural Heritage	2012	Archaeological excavation	Female	Am, PP, PPr, aDNA	Skeleton

Andong	DongGuk Institute of Cultural Properties	2013	Archaeological excavation	Male	Am, PP, PPr, CT, aDNA	Mummy

Dalsung	Gyeong-Sang Cultural Heritage Research Center	2014	Archaeological excavation	Female	Am, PP, PPr, CT, aDNA	Mummy

Junggye	Han Ul Research Institute of Cultural Heritage	2014	Archaeological excavation	Male	Am, PP, PPr	Skeleton

Daegu_HS	Daedong Institute of Cultural Heritage	2014	Archaeological excavation	Female	Am, PP, PPr, aDNA	Skeleton

Cheongdo	Yeongnam Institute of Cultural Properties	2015	Moving a grave	Male	Am, PP, PPr, CT, aDNA	Mummy

Yeongweol	Jungbu Institute for Archaeology	2015	Archaeological excavation	Male	Am, PP, PPr	Skeleton

Jangsung	Chungcheong Institue of Cultural Heritage	2017	Archaeological excavation	Male	Am, PP, PPr	Skeleton

Am, anthropometry; PP, paleopathology; PPr, paleoparasitology; CT, computed tomography; MRI, magnetic resonance imaging; aDNA, ancient DNA.

**Table 2:** Pathological findings observed during Korean mummy studies (until September 2018).

**Mummy**	**Estimated date**	**Pathological findings**
Yongin	15C-16C	*A. lumbricoides*, *T. trichiura*, *P. westermani*

Jinju	15C-16C	*A. lumbricoides*, *T. trichiura*, *P. westermani*

YG2-4	15C-16C	*A. lumbricoides*, *T. trichiura*, *P. westermani*

YG2-6	15C-16C	*A. lumbricoides*, *T. trichiura*, *P. westermani*

Andong	16C	Congenital Diaphragmatic Hernia, *T. trichiura*, *C. sinensis*

Sapgyo	16C	*T. trichiura*, *C. sinensis*, *M. yokogawai*, *G. seoi*

Hadong-1	17C	*C. sinensis*, *M. yokogawai*, *G. seoi*

Hadong-2	16C-17C	*P. westermanI *(Ectopic paragonimiasis)

Dalsung	16C-17C	*A. lumbricoides*, *T. trichiura*

Junggye	16C-17C	*A. lumbricoides*, *T. trichiura*, Taenia

Cheongdo	17C	*H. pylori, A. lumbricoides, P. westermani *(Liver abscess, ectopic paragonimiasis)

Sacheon	17C	*M. yokogawai*

Gangneung	17C	Lesion in the mandible, Calcified descending aorta,* T. trichiura*

Dangjin	17C	*H. pylori, A. lumbricoides*, *E. vermicularis*, *P. westermani*

Mungyeong	17C	Atherosclerosis, *M. tuberculosis T. trichiura*, *C. sinensis*

Waegwan	17C	*T. trichiura*, *C. sinensis*

PJ SM	17C	*A. lumbricoides*, *T. trichiura*

Seocheon	17C	*A. lumbricoides*, *T. trichiura*

Yangju	17C	Hepatitis B virus,* A. lumbricoides*, *T. trichiura*, *C. sinensis*

SN1-2	17C-18C	*T. trichiura*

SN3-7-1	16C-17C	*T. trichiura*

SN2-19-1	18C	*A. lumbricoides*, *T. trichiura*

SN2-19-2	18C	*A. lumbricoides*, *T. trichiura*

GJ1-2	17C-18C	*A. lumbricoides*, *T. trichiura*, *P. westermani*, *S. stercoralis*, *Trichostrongylus* spp., Taenia

Hwasung	18C	*A. lumbricoides*, *T. trichiura*, *P. westermani*

The full names of pathogens: *Ascaris lumbricoides; Trichuris trichiura; Enterobius vermicularis; Clonorchis sinensis; Paragonimus westermani; Metagonimus yokogawai; Gymnophalloides seoi; Strongyloides stercoralis; Helicobacter pylori; Mycobacterium tuberculosis*.

**Table 3:** Mummies of Warring States and Western Han Period, China.

Mummy	Year	Excavated Sites	Date of Death (or Estimated Period)	Sex	Archaeological findings
*Guo-Jia Gang Tomb No. 1*	1994	Jingmen city/ Hubei province	Middle stage of Warring States Period	Female	Duplicated coffin (1 outer; 1 inner); Well-preserved mummy

*Mawangdui*	1971	Changsha/ Hunan Province	After 168 BCE, but not too late	Female	Multiplicated coffin (2 outer; 4 inner); Charcoal and kaolin clay around the coffin; Water on the floor of the coffin; Well-preserved mummy

*Phoenix Hill No. 168*	1975	Jiangling County/ Hubei Province	167 BCE	Male	Triplicated coffin (1 outer; 2 inner); Clay around the coffin; Water on the floor of the coffin; Well-preserved mummy
